# Synthesis and antitumor activity of novel 2, 3-didithiocarbamate substituted naphthoquinones as inhibitors of pyruvate kinase M2 isoform

**DOI:** 10.1080/14756366.2017.1404591

**Published:** 2017-11-29

**Authors:** Xianling Ning, Hailong Qi, Ridong Li, Yan Jin, Michael A. McNutt, Yuxin Yin

**Affiliations:** a Institute of Systems Biomedicine, School of Basic Medical Sciences, Beijing Key Laboratory of Tumor Systems Biology, Peking University Health Science Center, Beijing, China;; b Department of Pharmacology, School of Basic Medical Sciences, Peking University Health Science Center, Beijing, China;; c Peking-Tsinghua Center for Life Sciences, Peking University Health Science Center, Beijing, China;; d Department of Pathology, School of Basic Medical Sciences, Peking University Health Science Center, Beijing, China

**Keywords:** M2 isoform of pyruvate kinase, PKM2 inhibitors, 2,3-didithiocarbamate substituted naphthoquinones, antiproliferative effects

## Abstract

The M2 isoform of pyruvate kinase (PKM2) is a potential antitumor therapeutic target. In this study, we designed and synthesised a series of 2, 3-didithiocarbamate substituted naphthoquinones as PKM2 inhibitors based on the lead compound **3k** that we previously reported. Among them, compound **3f** (IC_50_ = 1.05 ± 0.17 µM) and **3h** (IC_50_ = 0.96 ± 0.18 µM) exhibited potent inhibition of PKM2, and their inhibitory activities are superior to compound **3k** (IC_50_ = 2.95 ± 0.53 µM) and the known PKM2 inhibitor shikonin (IC_50_ = 8.82 ± 2.62 µM). In addition, we evaluated *in vitro* antiproliferative effects of target compounds using MTS assay. Most target compounds exhibited dose-dependent cytotoxicity with IC_50_ values in nanomolar concentrations against HCT116, MCF7, Hela, H1299 and B16 cells. These small molecule PKM2 inhibitors not only provide candidate compounds for cancer therapy, but also offer a tool to probe the biological effects of PKM2 inhibition on cancer cells.

## Introduction

The metabolism in cancer cells substantially differs from that in healthy cells^1–3^. The tumour cells rely on glycolysis to produce energy and have a high rate of glucose uptake but low rates of oxidative phosphorylation (Warburg effect)^4^^,^^5^. Cancer cells were found to have very strong metabolic dependencies, which are not associated with normal cells^6^. The interventions on tumour glycolysis become a novel strategy for selective anti-cancer therapies^7–9^.

Pyruvate kinase (PK) is the last rate-limiting enzyme in the glycolytic pathway and catalyses the transfer of a phosphate group from phosphoenolpyruvate (PEP) to adenosine diphosphate (ADP) to obtain pyruvate and adenosine triphosphate (ATP)^10^^,^^11^. There are M1, M2, L and R isoforms of PK in mammalian cells: the M1 isoform (PKM1) is expressed in many differentiated tissues (skeletal muscle, heart and brain), PKM2 is expressed during embryonic development or over expressed in tumour tissues, PKL and PKR are expressed in liver and erythrocytes, respectively^12–14^. PKM2 is important for cancer metabolism and tumour growth^15^^,^^16^. Tumourigenesis is associated with the re-expression of PKM2 together with a downregulation of the expression of PKM1 and other isozymes^17^^,^^18^. Therefore, targeting of PKM2 offers an opportunity to target cancer cell metabolism and reduce the side effects of cancer therapy^17^^,^^19^. However, an exact mechanistic understanding of PKM2 is still lacking. The identification of PKM2 inhibitors not only can provide candidate compounds for cancer therapy but also is helpful to deciphering other cellular functions of PKM2.

**Figure 1.**
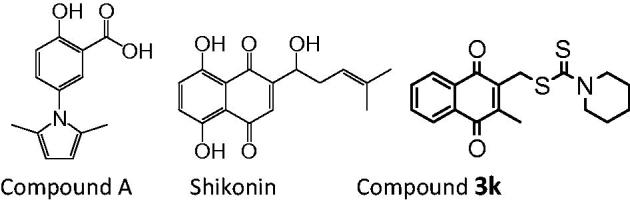
Structures of compound 3, shikonin and compound **3k**.

In 2010, Vander Heiden et al. screened a library of compounds to identify a small molecule PKM2 inhibitor compound A with IC_50_ value of 10 µM (Figure 1)^20^. Compound A was reported to presumably block the allosteric site of PKM2. In 2011, Chen et al. reported shikonin (Figure 1) and its enantiomeric isomer alkannin to inhibit PKM2 at low concentrations^21^^,^^22^. Recently, we reported a new PKM2 inhibitor compound **3k** (Figure 1) with IC_50_ value of 2.95 µM, which exhibits more potent PKM2 inhibitory activity than the known optimal PKM2 inhibitor shikonin (IC_50_ = 8.82 µM)^23^.

To further optimise this activity, in this study, some new analogues of **3k** were designed and synthesised. The PKM2 inhibitor compound **3f** and **3h** have high PKM2 inhibition responses, which are more potent than shikonin and compound **3k**, and they also exhibit high antiproliferative activity in several tumour cells.

## Materials and methods

### Chemistry

All chemicals, reagents and solvents were purchased from commercial sources. When necessary, they were purified and dried by standard methods. Reactions were checked by thin-layer chromatography (TLC) on pre-coated silica gel F_254_ plates. Column chromatography was carried out with Silica gel H (200–300 mesh or 500 mesh). Detection was by iodine vapour staining and UV light irradiation (UV lamp, model UV-IIB). Melting points were determined on an X_4_-type apparatus and are not corrected. ^1^H NMR and ^13^C NMR spectra were recorded on a Bruker AVANCE III-400 spectrometer, Chemical shifts δ in ppm with Me_4_Si as internal standard, coupling constants J in Hertz. High-resolution mass spectrum (HRMS) was recorded on a Thermo Scientific Orbitrap Elite MS.

### Procedure for preparation of 2,3-bis-chloromethyl-[1,4]naphthoquinone (2)^24^

The 1,4-naphthaquinone (**1**) (1 g, 6.3 mmol) in glacial acetic acid (20 ml) was taken in a 100-ml round-bottomed flask, and 36% aqueous formaldehyde (6 ml) was added. The reaction solution was cooled in ice water. Dry hydrogen chloride passed in for 2 h. The solution became red, then being kept at room temperature for 48 h. The reaction mixture was poured on ice and extracted with ethyl acetate. The combined organic fractions were washed with brine, dried (Na_2_SO_4_) and concentrated under reduced pressure. Purification of the crude residue by column chromatography (petroleum ether/ethyl acetate) afforded the compound **2** (yellow solid). The yield of this reaction was 68.9%. ^1^H NMR (400 MHz, CDCl_3_) *δ* 8.18–8.20 (m, 2H, Ar*H*), 7.81–7.83 (*m*, 2H, Ar*H*), 4.72 (*s*, 4H, 2CH*_2_*Cl).

### General procedure for preparation of dithiocarbamic acid 3-thiocarbamoylsulphanylmethyl-1,4-dioxo-1,4-dihydro-naphthalen-2-ylmethyl ester (3a-3h)

Carbon disulphide (180 μL, 3 mmol) and amine (3 mmol) were added to CH_3_CN (5 ml) and the resulting solution was stirred for 30 min. 2, 3-Bis-chloromethyl-[1,4]naphthoquinone (**2**) (254 mg, 1 mmol) was added in portions at frequent intervals. Then, the reaction mixture was kept at room temperature for 48 h. The reaction mixture was concentrated *in vacuo*, diluted with H_2_O, and extracted with CH_2_Cl_2_. The combined organic fractions were washed with brine, dried (Na_2_SO_4_) and concentrated under reduced pressure. Purification of the crude residue by column chromatography (petroleum ether/CH_2_Cl_2_) afforded the title compound.

*Data for dimorpholine-dithiocarbamic acid 3-dimorpholinethiocarbamoylsulphanylmethyl-1,4-dioxo-1,4-dihydro-naphthalen-2-ylmethyl ester (****3a****):* yellow solid (78.4%); mp 153–154 °C. ^1^H NMR (400 MHz, CDCl_3_) *δ* 8.11–8.13 (*m*, 2H, Ar*H*), 7.75–7.77 (*m*, 2H, Ar*H*), 4.89 (*s*, 4H, 2C*H_2_*S), 3.99–4.28 (*m*, 8H, 4OC*H_2_*), 3.76 (*s*, 8H, 4NC*H_2_*). ^13^C NMR (100 MHz, CDCl_3_) *δ* 196.1, 183.9, 143.7, 134.0, 132.0, 126.7, 66.3, 66.2, 34.0. HR-MS (ESI^+^) *m*/*z*: 509.0697 [M + H]^+^. Found: 509.0686 [M + H]^+^.

*Data for dimethyl-dithiocarbamic acid 3-dimethylthiocarbamoylsulphanylmethyl-1,4-dioxo-1,4-dihydro-naphthalen-2-ylmethyl ester (****3b****):* yellow solid (92.0%); mp 157–158 °C. ^1^H NMR (400 MHz, CDCl_3_) *δ* 8.11–8.13 (*m*, 2H, Ar*H*), 7.74–7.76 (*m*, 2H, Ar*H*), 4.83 (*s*, 4H, 2C*H_2_*S), 3.56 (*s*, 3H, NC*H_3_*), 3.36 (*s*, 3H, NC*H_3_*). ^13^C NMR (100 MHz, CDCl_3_) *δ* 195.7, 183.9, 143.8, 133.9, 132.0, 126.7, 45.7, 41.5, 34.1. HR-MS (ESI^+^) *m*/*z*: 425.0486 [M + H]^+^,447.0305 [M + Na]^+^. Found: 425.0487 [M + H]^+^, 447.0304 [M + Na]^+^.

*Data for diethyl-dithiocarbamic acid 3-diethylthiocarbamoylsulphanylmethyl-1,4-dioxo-1,4-dihydro-naphthalen-2-ylmethyl ester (****3c****):* yellow solid (94.3%); mp 130–131 °C. ^1^H NMR (400 MHz, CDCl_3_) *δ* 8.12–8.14 (*m*, 2H, Ar*H*), 7.74–7.76 (*m*, 2H, Ar*H*), 4.83 (*s*, 4H, 2C*H_2_*S), 4.03 (*q*, 4H, 2NC*H_2_*), 3.72 (*m*, 4H, 2NC*H_2_*), 1.29 (*m*, 12H, 4CH*_3_*). ^13^C NMR (100 MHz, CDCl_3_) *δ* 194.2, 183.9, 144.0, 133.8, 132.1, 126.7, 49.9, 46.8, 34.0, 12.6, 11.6. HR-MS (ESI^+^) *m*/*z*: 481.1112 [M + H]^+^, 503.0931 [M + Na]^+^. Found: 481.1111 [M + H]^+^, 503.0934 [M + Na]^+^.

*Data for dipropyl-dithiocarbamic acid 3-dipropylthiocarbamoylsulphanylmethyl-1,4-dioxo-1,4-dihydro-naphthalen-2-ylmethyl ester (****3d****):* yellow solid (92.9%); mp 111–112 °C. ^1^H NMR (400 MHz, CDCl_3_) *δ* 8.12–8.14 (*m*, 2H, Ar*H*), 7.73–7.76 (*m*, 2H, Ar*H*), 4.80 (*s*, 4H, 2C*H_2_*S), 3.91 (*q*, 4H, 2NC*H_2_*), 3.60 (*m*, 4H, 2NC*H_2_*), 1.73–1.79 (m, 8H, 4CH*_2_*CH_3_), 0.93–0.95 (*m*, 12H, 4C*H_3_*). ^13^C NMR (100 MHz, CDCl_3_) *δ* 194.7, 183.8, 144.1, 133.8, 132.1, 126.7, 57.3, 54.5, 34.1, 20.8, 19.6, 11.2. HR-MS (ESI^+^) *m*/*z*: 537.1738 [M + H]^+^. Found: 537.1728 [M + H]^+^, 559.1567 [M + Na]^+^.

*Data for diallyl-dithiocarbamic acid 3-diallylthiocarbamoylsulphanylmethyl-1,4-dioxo-1,4-dihydro-naphthalen-2-ylmethyl ester (****3e****):* yellow liquid (83.3%); ^1^H NMR (400 MHz, CDCl_3_) *δ* 8.11–8.14 (*m*, 2H, Ar*H*), 7.74–7.76 (*m*, 2H, Ar*H*), 5.80–5.91 (*m*, 4H, 4C*H*=CH_2_), 5.27 (d, 2H, CH=C*H*_2_), 5.25 (d, 2H, CH=C*H*_2_), 5.24 (d, 2H, CH=C*H*_2_), 5.20 (d, 2H, CH=C*H*_2_), 4.83 (*s*, 4H, 4C*H_2_*S), 4.66 (d, 4H, 2NC*H_2_*), 4.30 (d, 4H, 2NC*H_2_*). ^13^C NMR (100 MHz, CDCl_3_) *δ* 196.6, 183.8, 143.9, 133.9, 132.0, 131.0, 130.3, 126.7, 118.9, 118.7, 56.9, 53.8, 34.4. HR-MS (ESI^+^) *m*/*z*: 529.1112 [M + H]^+^. Found: 529.1113 [M + H]^+^.

*Data for dithiamorpholine-dithiocarbamic acid 3-dithiamorpholinethiocarbamoylsulphanylmethyl-1,4-dioxo-1,4-dihydro-naphthalen-2-ylmethyl ester (****3f****):* yellow solid (90.7%); mp 146–147 °C. ^1^H NMR (400 MHz, CDCl_3_) *δ* 8.11–8.13 (*m*, 2H, Ar*H*), 7.74–7.77 (*m*, 2H, Ar*H*), 4.66 (*s*, 4H, 2C*H_2_*S), 4.57–4.58 (*m*, 4H, 2NC*H_2_*), 4.26–4.29 (*m*, 4H, 2NC*H_2_*), 2.76 (*s*, 8H, 4C*H_2_*S). ^13^C NMR (100 MHz, CDCl_3_) *δ* 195.3, 183.9, 143.8, 134.0, 131.9, 126.7, 34.7, 27.3. HR-MS (ESI^+^) *m*/*z*: 541.0240 [M + H]^+^, 563.0060 [M + Na]^+^. Found: 541.0343 [M + H]^+^,563.0178 [M + Na]^+^.

*Data for dipyrrolidine-dithiocarbamic acid 3-dipyrrolidinethiocarbamoylsulphanylmethyl-1,4-dioxo-1,4-dihydro-naphthalen-2-ylmethyl ester (****3g****):* yellow solid (88.2%); mp 150–151 °C. ^1^H NMR (400 MHz, CDCl_3_) *δ* 8.11–8.13 (*m*, 2H, Ar*H*), 7.73–7.75 (*m*, 2H, Ar*H*), 4.88 (*s*, 4H, 2C*H_2_*S), 3.95 (*q*, 4H, 2NC*H_2_*), 3.64 (*q*, 4H, 2NC*H_2_*), 1.96–2.09 (*m*, 8H, 2CH_2_CH_2_). ^13^C NMR (100 MHz, CDCl_3_) *δ* 191.3, 184.0, 143.9, 133.9, 132.0, 126.6, 55.3, 50.6, 33.5, 26.2, 24.3. HR-MS (ESI^+^) *m*/*z*: 477.0799 [M + H]^+^, 499.0618[M + Na]^+^. Found: 477.0791 [M + H]^+^, 499.0626[M + Na]^+^.

*Data for dithiazolidine-dithiocarbamic acid 3-dithiazolidinethiocarbamoylsulphanylmethyl-1,4-dioxo-1,4-dihydro-naphthalen-2-ylme-thyl ester (****3h****):* yellow solid (88.8%); mp 158–159 °C. ^1^H NMR (400 MHz, CDCl_3_) *δ* 8.11–8.14 (*m*, 2H, Ar*H*), 7.75–7.77 (*m*, 2H, Ar*H*), 4.86 (*s*, 4H, 2C*H_2_*S), 3.11–5.04 (*m*, 12H, 2NC*H_2_*C*H_2_*SC*H_2_*). ^13^C NMR (100 MHz, CDCl_3_) *δ* 192.5, 183.9, 143.6, 134.0, 131.9, 126.7, 56.6, 52.7, 34.2, 31.2, 29.1. HR-MS (ESI^+^) *m*/*z*: 512.9927 [M + H]^+^, 534.9747 [M + Na]^+^. Found: 512.9944 [M + H]^+^, 534.9720 [M + Na]^+^.

### Biological activity

#### Purification of recombinant pyruvate kinase isoforms

Human cDNA for PKM2 was cloned into pET28a^+^ with a N-terminal His tag and purified from Escherichia coli strain BL21 (Invitrogen) using Ni-Agarose beads (Qiagen) as described previously^25^.

#### PKM2 activity assay

Pyruvate kinase activity was measured with a fluorescent pyruvate kinase-lactate dehydrogenase coupled assay as previously described^20^.

#### Cell culture

Cell lines were grown with routine culture techniques in RPMI 1640 supplemented with 9% foetal bovine serum at 37 °C in 5% CO_2_.

#### MTS cell proliferation assay

Cells were plated in 96-well plates at a density of 5000 cells per well. Twelve hours after seeding, cells were treated with various concentrations of test compounds for 48 h. Cell viability was assessed with the MTS assay (Promega) according to the manufacturer's instruction.

## Results and discussion

### Chemistry

**Scheme 1.**
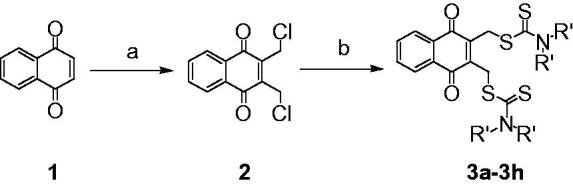
Synthesis of 2,3-didithiocarbamate-substituted naphthoquinones. Reagents and conditions: (a) formaldehyde, HCl, HAc, H_2_O, 0 °C, 68.9%; (b) CS_2_, amine, CH_3_CN, rt, 78–94%.

2, 3-Didithiocarbamate-substituted naphthoquinones (**3a**-**3h**) were prepared as target compounds as shown in Scheme 1. 1,4-Naphthoquinone **1** was reacted with formaldehyde in the presence of dry hydrogen chloride in a cold mixed solvent of H_2_O and acetic acid to provide 2,3-dichloromethyl-1,4-naphthoquinone **2**. Compound **2** was treated with CS_2_ and various amines to obtain target compounds **3a-3h** in moderate to high yields.

### Biological activity

#### PKM2 inhibition activity of compounds 3a-3h

We first tested the effect of compound **3a**-**3h** on PKM2 activity using a fluorescent PK-LDH coupled assay according to a previously reported method^20^. Shikonin, which is a known inhibitor of PKM2, was used as the positive control. Most of the target compounds exhibited some degree of PKM2 inhibitory activity. Compound **3f** (IC_50_ = 1.05 ± 0.17 µM) and **3h** (IC_50_ = 0.96 ± 0.18 µM) displayed the higher inhibitory activity than the lead compound **3k** (IC_50_ = 2.95 ± 0.53 µM) and shikonin (IC_50_ = 8.82 ± 2.62 µM) (Table 1). The preliminary SAR can be summarised as follows. The amine moiety of target compounds greatly influenced PKM2 inhibitory activity. Introduction of a short chain N, N-dimethylamine reduced the inhibitory activity (**3b** vs. **3c** and **3d**). When the n-propyl amine in **3d** was replaced by allyl amine (**3e**), the inhibitory activity was lowered. The thiazolidinyl **3h** (IC_50_ = 0.96 ± 0.18 µM) and thiamorpholinyl **3f** (IC_50_ = 1.05 ±  0.17 µM) substituted compounds respectively demonstrated stronger activity than pyrrolidinyl **3g** (IC_50_ = 2.33 ± 0.52 µM) and morpholinyl **3a** (IC_50_ = 2.64 ± 0.98 µM) substituted compounds. Introduction of a sulphur atom therefore contributed to improvement of PKM2 inhibitory activity.

**Table 1.** PKM2 inhibitory activity of compounds **3a**-**3h**.


Compound	*R*	IC_50_ ± SD (μM)
**3a**	morpholinyl	2.64 ± 0.98
**3b**	dimethylamino	>10
**3c**	diethylamino	2.78 ± 0.97
**3d**	di-n-propylamino	3.08 ± 1.23
**3e**	diallylamino	>10
**3f**	thiamorpholinyl	1.05 ± 0.17
**3g**	pyrrolidinyl	2.33 ± 0.52
**3h**	thiazolidinyl	0.96 ± 0.18
**3k**		2.95 ± 0.53
**Shikonin**		8.82 ± 2.62

#### Antiproliferative effects of target compounds 3a-3h

To determine the efficiency of **3a**-**3h** as antitumour agents, we assessed the *in vitro* cytotoxicity of **3a**-**3h** using several different tumour cell lines derived from human colon cancer (HCT116), breast cancer (MCF7), cervical cancer (Hela) and lung cancer (H1299) and mouse melanoma (B16). The results are presented in Table 2. Most target compounds reduced cancer cell viability at nanomolar concentrations in MTS reduction assays, showing higher cytotoxicity than shikonin. Specially, compound **3b** exhibited an optimal dose-dependent cytotoxicity with IC_50_ values against HCT116, MCF7, Hela, H1299 and B16 cells from 69 nM to 122 nM. The preliminary SAR showed that introduction of a long-chain amine in target compounds lowered cytotoxicity (**3b** vs. **3c** vs **3d**), which was not consistent with the enzyme activity. This discrepancy may be due to the different properties of these compounds such as cell penetration that is important in the cellular assay. In addition, replacing the chain amines with various cyclic amines, morpholinyl (**3a**), thiamorpholinyl (**3f**), pyrrolidinyl (**3g**) and thiazolidinyl (**3h**) substitution compounds also demonstrated the great potency.

**Table 2.** *In vitro* cytotoxicity of target compounds

	IC_50_ ± SD^a^ (μM)				
compd	HCT116	MCF7	Hela	H1299	B16
**3a**	0.214 ± 0.003	0.340 ± 0.003	0.337 ± 0.054	0.331 ± 0.091	0.272 ± 0.010
**3b**	0.088 ± 0.004	0.069 ± 0.009	0.122 ± 0.017	0.109 ± 0.002	0.104 ± 0.011
**3c**	0.093 ± 0.002	0.084 ± 0.003	0.251 ± 0.059	0.144 ± 0.013	0.108 ± 0.010
**3d**	0.597 ± 0.014	>10	>10	0.830 ± 0.162	0.787 ± 0.203
**3e**	0.742 ± 0.045	1.092 ± 0.421	3.456 ± 3.188	1.019 ± 0.111	0.709 ± 0.027
**3f**	0.189 ± 0.035	0.194 ± 0.055	0.412 ± 0.018	0.206 ± 0.018	0.189 ± 0.006
**3g**	0.164 ± 0.003	0.638 ± 0.020	0.298 ± 0.053	0.247 ± 0.015	0.159 ± 0.002
**3h**	0.374 ± 0.015	0.234 ± 0.045	1.126 ± 0.080	0.306 ± 0.008	0.317 ± 0.022
**Shikonin**	1.060 ± 0.182	2.405 ± 0.346	0.813 ± 0.081	0.954 ± 0.186	1.220 ± 0.155

To further explore the selectivity of target compounds against cancer cells, we tested the cytotoxicity of representative compound **3f** in BEAS-2B cells derived from normal human bronchial epithelial cells. The result showed that IC_50_ value of compound **3f** was 11.49 ± 0.62 µM, which indicated the target compound had higher selectivity for cancer cells than normal cells.

## Conclusions

We have described the identification and characterisation of a previously undescribed chemical class of PKM2 inhibitors. Most target compounds as PKM2 inhibitors, especially compound **3f** and **3h**, have high inhibition responses and are more potent than shikonin and compound **3k**. These compounds are therefore optimal PKM2 inhibitors with the best characteristics reported to date. They may lock PKM2 into a low activity conformation and forces disruption of cancer cell metabolism in a manner that is less metabolically flexible than the normal state.

In addition, we have investigated *in vitro* cytotoxicity of these PKM2 inhibitors. Most target compounds show higher antitumour effects than shikonin in MTS assay. The compound **3b** and **3c** exhibited optimal dose-dependent cytotoxicity with IC_50_ values against HCT116, MCF7, Hela, H1299 and B16 cells, respectively, from 69 nM to 122 nM and from 84 nM to 251 nM.

However, there is absence of correlation between the PKM2 inhibitory activity and *in vitro* antitumor activity of the target compounds. This suggests that these compounds may have other mechanisms to influence the tumour cells. In future studies, we will focus on evaluation as yet unidentified mechanisms of these compounds.
